# Severity of subjective forgetfulness is associated with high dietary intake of copper in Japanese senior women: A cross‐sectional study

**DOI:** 10.1002/fsn3.1740

**Published:** 2020-07-01

**Authors:** Tamami Odai, Masakazu Terauchi, Risa Suzuki, Kiyoko Kato, Asuka Hirose, Naoyuki Miyasaka

**Affiliations:** ^1^ Department of Obstetrics and Gynecology Tokyo Medical and Dental University Bunkyo Tokyo Japan; ^2^ Department of Women’s Health Tokyo Medical and Dental University Bunkyo Tokyo Japan

**Keywords:** cognitive function, dietary nutrient intake, memory, menopausal symptoms, mineral

## Abstract

This study investigated the relationship between subjective forgetfulness and the dietary intake of various nutrients in middle‐aged and senior women. A cross‐sectional study of the first‐visit records of 245 Japanese women aged 40 or over was performed. The severity of subjective forgetfulness was classified according to the Menopausal Health‐Related Quality of Life Questionnaire: none and mild (“unforgetful”) or moderate and severe (“forgetful”). Dietary consumption of nutrients was estimated using the brief‐type self‐administered diet history questionnaire. The associations between the severity of subjective forgetfulness and intake of 43 major nutrients were evaluated using multivariate logistic regression analysis separately performed for two age groups: middle‐aged (40–54 years, *N* = 166) and senior (55 years or over, *N* = 79). No nutrients were found to be significantly associated with subjective forgetfulness in the middle‐aged group. In senior women, a significant positive relationship between the intake of copper and forgetfulness was found (adjusted odds ratio per 10 mg/kJ increase in copper intake: 1.25; 95% confidence interval: 1.08–1.50). Thus, high copper intake is positively associated with the severity of forgetfulness in Japanese senior women. Reducing copper consumption could help improve this symptom in this population.

## Graphical abstract

We examined the nutritional factors associated with subjective forgetfulness. Participants were Japanese middle‐aged and elderly women. High copper intake is positively associated with the severity of forgetfulness in Japanese senior women.

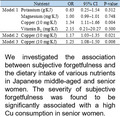

## 1 INTRODUCTION

Cognitive functions such as memory, thought, learning, and decision‐making decline with age in a linear or quadratic fashion (Baltes & Lindenberger, 1997; Park et al., 1996). According to a worldwide report, 46.8 million people suffer from dementia, and this number is estimated to reach 131.5 million in 2050 (Alzheimer’s Disease International, 2015). In particular, the number of newly diagnosed cases of dementia in Asia is growing rapidly, currently accounting for approximately 50% of the global incidence (Alzheimer’s Disease International, 2015). Furthermore, the incidence of Alzheimer’s disease (AD) is higher in women than men, which is likely associated with estrogen depletion in the postmenopausal period, as estrogen regulates neurotrophin signaling and neurotransmission and protects against oxidative stress and inflammation in the brain (Brann, Dhandapani, Wakade, Mahesh, & Khan, 2007; Gurvich, Hoy, Thomas, & Kulkarni, 2018; Markou, Duka, & Prelevic, 2005; Viña & Lloret, 2010). Several studies have shown that the menstrual period affects verbal memory (Rosenberg & Park, 2002), with high estradiol levels being associated with good cognitive function (Gholizadeh, Sadatmahalleh, & Ziaei, 2018). Additionally, there are several reports that peri‐ and postmenopausal women have significantly more memory problems than premenopausal women. For example, subjective memory decline was observed in 62%–70% of postmenopausal women (Betti et al., 2001; Sullivan Mitchell & Fugate Woods, 2001), and the prevalence of subjective memory complaints in peri‐ and postmenopausal women was over three times greater than that in premenopausal women (Devi, Hahn, Massimi, & Zhivotovskaya, 2005).

Combined with the fear of the adverse effects of hormone therapies, such as cancer and cardiovascular disease, there is growing demand for nutraceutical supplements that could improve cognitive factors. Nutrients such as vitamins, trace elements, amino acids, and fatty acids are essential for brain structure and function (Bourre, 2006a, 2006b; Fernstrom, 1669). For example, vitamin B_1_ plays a key role in brain energy metabolism via glucose consumption; its deficiency causes peripheral and central nervous system disorders, such as the well‐known beriberi and Wernicke‐Korsakoff syndromes (Bourre, 2006a). A lack of zinc, which has neuromodulatory properties, is attributed to memory and learning impairment (Bourre, 2006a). Impaired copper (Cu) homeostasis in the brain is associated with neurodegeneration, such as Menkes disease and Wilson’s disease (Scheiber, Mercer, & Dringen, 2014). Lowered consumption of tyrosine, an essential amino acid, and a precursor for catecholamine neurotransmitters including dopamine, a regulator of cognitive function (Previc, 1999), is associated with cognitive dysfunction (Hensel et al., 2019). Docosahexaenoic acid, a known n‐3 fatty acid component of the myelin involved in signal transduction, has also been reported to enhance cognitive function (Cole & Frautschy, 2010). Moreover, there are several reports on the effect of nutritional supplements, including vitamin E and n‐3 fatty acids, on cognitive function in elderly adults, mild cognitive impairment, and AD (Farina, Llewellyn, Isaac, & Tabet, 2017; Fraga, Carvalho, Caramelli, de Sousa, & Gomes, 2017). However, the effects of the dietary intake of nutrients on memory in the peri‐ and postmenopausal periods have not yet been fully elucidated.

In the present study, we investigated the associations between the severity of subjective forgetfulness and the dietary consumption of a variety of nutrients in Japanese middle‐aged and senior women.

## 2 MATERIALS AND METHODS

### 2.1 Study population

In this cross‐sectional study, we analyzed the first‐visit medical records of 700 Japanese women enrolled in the Systematic Health and Nutrition Education Program conducted at the menopause clinic of Tokyo Medical and Dental University from January 2009 to August 2017. This program aimed to promote the psychological and physical health of women according to a comprehensive assessment of general physical and mental health status and lifestyle via physical examinations and various questionnaires. All the women who participated in this program visited the clinic for treatment of menopausal symptoms. Of the 700 participants, 382 women aged 40 or over completed both the Menopausal Health‐Related Quality of Life Questionnaire (MHR‐QOL) and the brief‐type self‐administered diet history questionnaire (BDHQ), evaluating the severity of subjective forgetfulness and dietary habits, respectively. Thirty‐nine participants whose menopausal status was unclear due to a hysterectomy (and unilateral oophorectomy), 57 participants who had received hormone therapy, and 41 participants who had received psychotropic drugs such as hypnotics, antidepressants, and anxiolytics were excluded. For the remaining 245 participants, we investigated the associations between the severity of subjective forgetfulness and their dietary consumption of various nutrients.

The research protocol was reviewed and approved by the Tokyo Medical and Dental University Review Board, and written informed consent was obtained from all participants. All study procedures were conducted in accordance with the Declaration of Helsinki.

### 2.2 Measurements

#### 2.2.1 Menopausal status

The women were classified as pre‐ or postmenopausal if they had a regular period or no menstruation in the past 12 months, respectively. Perimenopausal status was assigned to those who had a menstruation within the past 12 months but had missed periods or had had an irregular cycle during the past 3 months.

#### 2.2.2 Physical assessments

The body composition of participants, including height, weight, body mass index (BMI), fat mass, lean body mass, muscle mass, water mass, basal metabolism, and visceral fat level, was measured using a body composition analyzer (MC190‐EM; Tanita). The participants’ waist and hip circumferences were measured, and their waist‐to‐hip ratios were evaluated. Resting energy expenditure was calculated based on respiratory volume using an indirect calorimeter (Metavine‐V VMB‐005N; Vine). Their body temperature was measured with a thermometer. We also assessed cardiovascular parameters such as blood pressure, heart rate, cardio‐ankle vascular index, and ankle‐brachial pressure index (VS‐1000; Fukuda Denshi). Physical fitness tests for power, reaction time, and flexibility were also conducted. Hand‐grip strength was measured twice for each hand with a hand dynamometer (Yagami) to calculate average hand‐grip strength (kgf) using the larger value from each hand. The ruler drop test was performed to evaluate reaction time using a wooden ruler 60 cm in length and weighing 110 g (Yagami). Seated participants, fixing their arms on a desk and outstretching their fingers from the edge of the desk, attempted to catch the ruler, whose bottom was hung between their thumbs and index fingers by an examiner, as quickly as possible when it was dropped. The scale on the ruler was used to measure where the participants gripped. After the test was repeated seven times and the largest and smallest values were omitted, the average reaction time (cm) was calculated from the remaining five values. The sit‐and‐reach test as an assessment of flexibility was performed using a reach box while sitting (Yagami).

#### 2.2.3 Lifestyle characteristics

Lifestyle factors such as frequency of smoking (none, fewer than 20, more than 20 cigarettes per day), consumption of alcohol (never, sometimes, daily) and caffeinated drinks (none, once or twice, three or more times per day), and regular exercise habits (yes, no), were assessed via a medical interview.

#### 2.2.4 Questionnaire

The MHR‐QOL (Table S1) comprises four domains: physical health, mental health, life stratification, and social involvement. It is a modification of the Women’s Health Questionnaire and others (Hanson, Isacsson, Janzon, & Lindell, 1989; Hunter, Battersby, & Whitehead, 1986; van Keep & Kellerhals, 1974) that was developed and validated in our clinic (Hirose et al., 2016; Terauchi et al., 2010, 2011, 2012, 2015; Terauchi, Hirose, Akiyoshi, Kato, & Miyasaka, 2017). Its 4‐point Likert scale for physical and psychological symptoms is based on symptom frequency. Respondents indicate the degree of agreement or disagreement with statements about life satisfaction and social involvement on a 2‐ or 4‐point Likert scale. According to the participants’ responses, the severity of their subjective forgetfulness was rated as none, mild, moderate, or severe based on frequency (none, zero to one time per month; mild, one to two times per week; moderate, three to four times per week; severe, almost every day). In the present study, those with no or mild forgetfulness were defined as “unforgetful” and those with moderate or severe forgetfulness as “forgetful.” We added up the scores for somatic symptoms (six items), vasomotor symptoms (two items), insomnia symptoms (two items), life satisfaction (five items), and social involvement (12 items) to produce a score for each subcategory, with a high score representing good health and a high level of satisfaction and social engagement.

The BDHQ was developed to assess the intake frequency over the previous month of 61 food items in the typical Japanese diet, including beverages and seasonings. Based on the information provided in response, the daily consumption of 96 nutrients, after adjustment for total calorie intake, was estimated by an ad hoc computer algorithm. The BDHQ was validated by comparison with dietary records using a semi‐weighed method (Kobayashi et al., 2011, 2012). In the present study, we focused on 43 major nutrients with high validity (Table S2).

The Hospital Anxiety and Depression Scale (HADS), a reliable screening instrument for anxiety and depression, was developed to assess the psychological health of patients with physical symptoms (Zigmond & Snaith, 1983). The HADS contains seven items each for anxiety and depression, and participants respond to these items on a 4‐point Likert scale. Participants who scored 8–10 were considered to likely be experiencing anxiety or depression, while those who scored 11–21 were considered to definitely be experiencing anxiety or depression.

### 2.3 Statistical analysis

Continuous variables are presented as mean ± standard deviation. The required sample size was estimated at 250, calculated as 10 times the number of independent variables, estimated at 10, divided by the prevalence of forgetfulness, estimated at 0.4. The Kruskal–Wallis test, Mann–Whitney test, and chi‐squared test were used to evaluate the difference between groups. Multicollinearity between variables was identified using cutoff points for Pearson or Spearman correlation coefficients of |*R*| > .9. The relationship between the severity of subjective forgetfulness and daily consumption of nutrients was analyzed using multivariate logistic regression analysis after adjustment for age, BMI, and the background factors related to forgetfulness obtained through the analysis.* p* values of <.05 were considered statistically significant. All statistical analyses were performed with GraphPad Prism version 5.02 (GraphPad Software) and JMP version 12 (SAS Institute Inc).

## 3 RESULTS

The average age of the 245 study participants was 53.4 ± 6.8 years (mean ± standard deviation). The percentages of women with none, mild, moderate, and severe subjective forgetfulness were 33.5%, 35.5%, 15.9%, and 15.1%, respectively (Table 1). The relevant background characteristics are presented in Table 2. The forgetful participants complained of physical and psychological symptoms such as somatic and vasomotor problems, insomnia, anxiety, and depression more frequently than the unforgetful ones, and their life satisfaction was significantly lower. We first assessed the nutrients whose intake differed significantly between the four groups of subjective forgetfulness severity, finding no nutrient to be significantly associated with forgetfulness in the whole group.

**Table 1:** Percentage of women bothered by forgetfulness

Frequency of forgetfulness	All participants (*N* = 245)		Middle‐aged (*N* = 166)		Senior (*N* = 79)	
	*n*	%	*n*	%	*n*	%
0–1 times a month (none)	82	33.5	56	33.7	26	32.9
1–2 times a week (mild)	87	35.5	57	34.3	30	38.0
0–3 times a week (moderate)	39	15.9	28	16.9	11	13.9
Almost every day (severe)	37	15.1	25	15.1	12	15.2

**Table 2:** Comparison of participant characteristics between the severity categories of subjective forgetfulness

	None (*N* = 82)	Mild (*N* = 87)	Moderate (*N* = 39)	Severe (*N* = 37)	*p*‐Value
Age, years	52.9 (6.9)	53.7 (6.5)	54.0 (7.0)	53.1 (7.1)	.615^a^
Menopausal status, %					
Pre/peri/postmenopausal	28.0/12.2/59.8	23.0/18.4/58.6	25.6/20.5/53.9	29.7/16.2/54.1	.880^b^
Body composition					
Height, cm	157.7 (6.0)	156.6 (5.9)	155.2 (6.4)	157.4 (4.9)	.138^a^
Weight, kg	54.7 (9.1)	54.3 (9.8)	54.6 (10.7)	53.8 (9.4)	.989^a^
Body mass index, kg/m^2^	22.0 (3.5)	22.2 (3.9)	22.6 (3.7)	21.7 (3.4)	.602^a^
Waist–hip ratio	0.88 (0.06)	0.88 (0.06)	0.88 (0.06)	0.87 (0.07)	.579^a^
Fat mass, kg	15.9 (6.6)	16.1 (7.3)	16.4 (7.5)	15.4 (6.4)	.951^a^
Lean mass, kg	38.8 (3.7)	38.2 (3.5)	38.2 (3.9)	38.4 (3.9)	.614^a^
Muscle mass, kg	36.5 (3.4)	36.0 (3.2)	36.0 (3.6)	36.2 (3.6)	.623^a^
Water mass, kg	28.0 (3.4)	27.6 (3.2)	27.9 (3.4)	27.7 (3.6)	.909^a^
Basal metabolism, MJ/day	4.70 (0.52)	4.63 (0.51)	4.64 (0.59)	4.64 (0.55)	.723^a^
Viceral fat level	5.3 (2.7)	5.5 (2.8)	5.6 (3.0)	4.9 (2.4)	.783^a^
Resting energy expenditure, MJ/day	6.95 (1.85)	6.92 (1.98)	7.01 (1.86)	6.94 (1.55)	.980^a^
Body temperature, °C	36.0 (0.9)	36.1 (0.4)	36.2 (0.6)	36.2 (0.5)	.481^a^
Physical fitness test					
Hand‐grip strength, kg	26.5 (4.1)	24.8 (5.4)	24.5 (4.6)	24.8 (4.8)	.105^a^
Ruler drop test, cm	22.2 (4.0)	22.9 (4.1)	23.9 (4.6)	23.7 (4.2)	.166^a^
Toe touch test, cm	36.5 (10.7)	35.4 (9.8)	36.2 (7.2)	34.7 (12.3)	.759^a^
Cardiovascular parameters					
Systolic blood pressure, mmHg	125.8 (18.0)	127.4 (18.0)	126.3 (18.1)	122.2 (16.9)	.733^a^
Diastolic blood pressure, mmHg	76.2 (11.0)	76.0 (12.4)	74.8 (13.5)	73.0 (12.4)	.584^a^
Heart rate, min^−1^	78.0 (13.2)	79.2 (13.2)	78.2 (10.8)	75.3 (11.8)	.208^a^
Cardio‐ankle vascular index	7.62 (0.69)	7.54 (0.82)	7.70 (0.81)	7.54 (0.87)	.792^a^
Ankle‐brachial pressure index	1.11 (0.06)	1.11 (0.05)	1.11 (0.07)	1.11 (0.08)	.810^a^
Lifestyle factors					
Smoking, %					
None/fewer than 20/20 or more cigarettes per day	90.2/6.1/3.7	93.1/3.4/3.4	94.9/2.6/2.6	94.6/0.0/5.4	.764^b^
Drinking, %					
Never/sometimes/daily	69.5/22.0/8.5	62.1/28.7/9.2	64.1/25.6/10.3	62.2/27.0/10.8	.967^b^
Caffeine, %					
Never/1−2 times/3 or more times per day	11.0/31.7/57.3	10.5/26.7/62.8	10.3/43.6/46.2	5.4/37.8/56.8	.563^b^
Exercise, %					
Yes/no	53.7/46.3	51.2/48.8	51.3/48.7	35.1/64.9	.288^b^
Menopausal Health‐Related Quality of Life Questionnaire					
Somatic symptom score (0–18 points)	15.7 (3.8)	13.7 (3.4)	12.4 (4.3)	11.7 (3.7)	<.001^a^
Vasomotor symptom score (0–6 points)	4.4 (2.1)	3.6 (2.1)	3.4 (2.4)	3.7 (2.1)	.024^a^
Insomnia symptom score (0–6 points)	4.5 (1.9)	4.3 (1.8)	3.5 (2.1)	3.3 (2.2)	.002^a^
Life satisfaction score (0–15 points)	9.2 (3.6)	7.6 (2.9)	6.7 (3.8)	6.2 (3.0)	<.001^a^
Social involvement score (0–16 points)	9.9 (2.9)	9.3 (2.7)	9.4 (3.5)	8.9 (3.0)	.272^a^
Hospital Anxiety and Depression Scale	9.7 (5.6)	14.2 (5.5)	16.1 (6.6)	15.5 (7.2)	<.001^a^
Anxiety subscale score	5.3 (3.2)	7.4 (3.0)	8.2 (3.8)	7.8 (4.0)	<.001^a^
Depression subscale score	4.3 (3.1)	6.8 (3.3)	7.9 (3.4)	7.7 (3.9)	<.001^a^

Note

Values are mean (standard deviation) or percentage.

^a^ Kruskal–Wallis test.

^b^ Chi‐squared test.

Next, we performed a post hoc analysis of the 245 participants divided into two age‐based groups, middle‐aged (40–54 years, *N* = 166), and senior (55 years or over *N* = 79), and evaluated the relationship between severity of subjective forgetfulness and dietary intake of nutrients in each group. The rates of subjective forgetfulness and the characteristics of each group are shown in Tables 1 and 3, and Table S3. The senior group had a significantly higher waist‐to‐hip ratio and visceral fat level and lower basal metabolism and vascular health, while the middle‐aged group had more severe physical and mental symptoms and lower levels of satisfaction and social involvement (Table 3). Moreover, the senior women consumed significantly more nutrients (except the following 11: fat, animal and vegetable fat, carbohydrates, retinol, saturated fatty acids, monounsaturated fatty acids, polyunsaturated fatty acids, cholesterol, n‐6 fatty acids, and alcohol) than the middle‐aged women despite no difference between the groups in total energy intake (*p* = .174) (Table 4). No nutrient was significantly related to the severity of subjective forgetfulness in the middle‐aged group. However, in the senior group, the daily intake of potassium, magnesium, Cu, and vitamin B_1_ differed significantly among the four severity groups (potassium, *p* = .036; magnesium, *p* = .036; Cu, *p* = .016; vitamin B_1_, *p* = .029). Participants with more severe forgetfulness consumed more of these nutrients. There was no multicollinearity among these four nutrients. Next, we investigated the background factors that differed significantly between the four severity groups in senior women. The results showed that reaction time and the scores for somatic symptoms, life satisfaction, social involvement, anxiety, and depression were found to be related to forgetfulness. There was no multicollinearity between these background factors. We used these six background factors as independent variables. Multivariate logistic regression analysis was conducted to identify the association between the intake of selected nutrients (potassium, magnesium, Cu, and vitamin B_1_) and the severity of subjective forgetfulness. After adjustment for the selected four nutrients (Model 1), for age and BMI (Model 2), and for age, BMI, and the related background factors (Model 3), an association between higher dietary consumption of Cu (10 mg/kJ) and the severity of subjective forgetfulness in the senior group was found (Table 5; Model 2, adjusted odds ratio [AOR] = 1.17, CI = 1.03–1.35, *p* = .021; Model 3, AOR = 1.25, CI = 1.08–1.50, *p* = .006).

**Table 3:** Comparison of the characteristics of middle‐aged and senior women

	Middle‐aged (*N* = 166)	Senior (*N* = 79)	*p*‐Value
Age, years	49.6 (2.9)	61.4 (5.4)	<.001^a^
Menopausal status, %			
Pre/peri/postmenopausal	38.6/23.5/38.0	0.0/1.3/98.7	<.001^b^
Body composition			
Height, cm	157.5 (5.8)	155.5 (6.0)	.019^a^
Weight, kg	55.1 (10.0)	53.0 (8.6)	.212^a^
Body mass index, kg/m^2^	22.2 (3.8)	21.9 (3.2)	.805^a^
Waist–hip ratio	0.87 (0.06)	0.89 (0.06)	.005^a^
Fat mass, kg	16.4 (7.4)	15.1 (5.8)	.358^a^
Lean mass, kg	38.7 (3.6)	37.8 (3.8)	.161^a^
Muscle mass, kg	36.5 (3.3)	35.7 (3.5)	.161^a^
Water mass, kg	27.9 (3.3)	27.5 (3.3)	.295^a^
Basal metabolism, MJ/day	4.71 (0.53)	4.54 (0.52)	.035^a^
Viceral fat level	5.2 (2.9)	5.8(2.3)	.012^a^
Resting energy expenditure, MJ/day	7.03 (1.88)	6.78 (1.77)	.455^a^
Body temperature, °C	36.2 (0.7)	36.0 (0.5)	.003^a^
Physical fitness test			
Hand‐grip strength, kg	25.4 (5.0)	25.1 (4.3)	.432^a^
Ruler drop test, cm	22.8 (4.0)	23.2 (4.4)	.512^a^
Sit‐and reach test, cm	34.9 (9.8)	37.8 (10.5)	.021^a^
Cardiovascular parameters			
Systolic blood pressure, mmHg	125.9 (17.4)	125.9 (18.8)	.764^a^
Diastolic blood pressure, mmHg	76.0 (12.0)	74.1 (12.3)	.234^a^
Heart rate, min^−1^	79.4 (13.5)	75.3 (10.0)	.066^a^
Cardio‐ankle vascular index	7.41 (0.71)	8.00 (0.79)	<.001^a^
Ankle‐brachial pressure index	1.11 (0.06)	1.11 (0.07)	.898^a^
Lifestyle factors			
Smoking, %			
None/fewer than 20/20 or more cigarettes per day	92.2/4.8/3.0	93.7/1.3/5.1	.291^b^
Drinking, %			
Never/sometimes/daily	59.6/30.1/10.2	75.9/16.5/7.6	.039^b^
Caffeine, %			
Never/1−2 times/3 or more times per day	8.4/36.1/55.4	12.8/25.6/61.5	.205^b^
Exercise, %			
Yes/no	41.0/59.0	67.9/32.1	<.001^b^
Menopausal Health‐Related Quality of Life Questionnaire			
Somatic symptom score (0–18 points)	13.1 (4.0)	15.3 (3.6)	<.001^a^
Vasomotor symptom score (0–6 points)	3.5 (2.2)	4.5 (1.9)	<.001^a^
Insomnia symptom score (0–6 points)	3.9 (2.1)	4.5 (1.8)	.066^a^
Life satisfaction score (0–15 points)	7.2 (3.4)	9.1 (3.3)	<.001^a^
Social involvement score (0–16 points)	9.1 (2.9)	10.3 (2.8)	.003^a^
Hospital Anxiety and Depression Scale	14.3 (6.4)	11.0 (6.1)	<.001^a^
Anxiety subscale score	7.3 (3.5)	6.1 (3.5)	.013^a^
Depression subscale score	6.9 (3.6)	4.9 (3.2)	<.001^a^

Note

Values are mean (standard deviation) or percentage.

^a^ Mann–Whitney test

^b^ Chi‐squared test.

**Table 4:** Comparison of the daily intake of nutrients between the middle‐aged and senior female participants

Nutrients	Middle‐aged (*N* = 166)	Senior (*N* = 79)	*p*‐Value^a^
Total energy, MJ	6.95 (1.92)	7.27 (1.96)	.174
Protein, %E	15.5 (3.1)	17.6 (2.5)	<.001
Animal protein, %E	8.7 (3.2)	10.4 (2.8)	<.001
Vegetable protein, %E	6.8 (1.3)	7.2 (1.3)	.020
Carbohydrate, %E	52.8 (8.2)	52.4 (7.1)	.561
Soluble dietary fiber, g/MJ	0.46 (0.16)	0.56 (0.16)	<.001
Insoluble dietary fiber, g/MJ	1.25 (0.40)	1.53 (0.43)	<.001
Dietary fiber, g/MJ	1.76 (0.59)	2.17 (0.59)	<.001
Fat, %E	28.2 (5.7)	27.7 (4.9)	.502
Animal fat, %E	12.6 (4.1)	13.3 (3.9)	.165
Vegetable fat, %E	15.7 (3.8)	14.4 (3.4)	.009
Saturated fatty acid, %E	7.7 (1.9)	7.5 (2.0)	.120
Monounsaturated fatty acid, %E	10.0 (2.3)	9.6 (2.0)	.130
Polyunsaturated fatty acid, %E	6.8 (1.5)	6.8 (1.2)	.874
Cholesterol, mg/MJ	43.9 (15.3)	47.2 (15.1)	.054
n‐3 fatty acid, %E	1.3 (0.4)	1.5 (0.4)	<.001
n‐6 fatty acid, %E	5.4 (1.2)	5.3 (1.0)	.333
Ash, g/MJ	2.50 (0.47)	2.90 (0.54)	<.001
Sodium, mg/MJ	550.0 (111.0)	618.8 (146.5)	<.001
Potassium, mg/MJ	369.3 (104.0)	446.9 (94.7)	<.001
Calcium, mg/MJ	78.8 (27.2)	97.8 (29.9)	<.001
Magnesium, mg/MJ	35.4 (8.4)	41.8 (7.2)	<.001
Phosphorus, mg/MJ	142.3 (31.2)	164.9 (28.8)	<.001
Iron, mg/MJ	1.09 (0.28)	1.30 (0.24)	<.001
Zinc, mg/MJ	1.09 (0.18)	1.18 (0.15)	<.001
Copper, mg/MJ	0.15 (0.03)	0.17 (0.02)	<.001
Manganese, mg/MJ	0.42 (0.15)	0.49 (0.13)	<.001
Retinol, μg/MJ	53.1 (31.7)	53.8 (29.7)	.551
β‐Carotene, μg/MJ	582.2 (375.2)	768.9 (400.1)	<.001
Retinol equivalent, μg/MJ	101.9 (46.7)	118.2 (38.9)	.002
Vitamin D, μg/MJ	1.66 (1.12)	2.41 (1.41)	<.001
α‐Tocopherol, mg/MJ	1.05 (0.27)	1.17 (0.23)	<.001
Vitamin K, μg/MJ	49.0 (25.6)	60.9 (28.3)	.001
Vitamin B_1_, mg/MJ	0.11 (0.02)	0.12 (0.02)	<.001
Vitamin B_2_, mg/MJ	0.19 (0.05)	0.21 (0.04)	<.001
Niacin, mgNE/MJ	2.30 (0.61)	2.61 (0.51)	<.001
Vitamin B_6_, mg/MJ	0.17 (0.04)	0.21 (0.04)	<.001
Vitamin B_12_, μg/MJ	1.14 (0.54)	1.51 (0.64)	<.001
Folic acid, μg/MJ	49.0 (18.7)	61.7 (18.4)	<.001
Pantothenic acid, mg/MJ	0.90 (0.1)	1.01 (0.15)	<.001
Vitamin C, mg/MJ	16.9 (7.7)	22.8 (7.6)	<.001
Daidzein, mg/MJ	2.01 (1.36)	2.44 (1.16)	.006
Genistein, mg/MJ	3.50 (2.29)	4.13 (1.95)	.005
Alcohol, %E	2.7 (6.1)	2.0 (5.7)	.001

Note

Values are mean (standard deviation).

Abbreviation: NE, niacin equivalent.

^a^ Mann–Whitney test.

**Table 5:** Relationship between daily intake of copper (10 mg/kJ) acid and severity of subjective forgetfulness

	Nutrient	OR	95% CI	*p*‐Value
Model 1	Potassium (g/kJ)	0.63	0.25–1.54	.312
	Magnesium (mg/kJ)	1.00	0.99–1.01	.748
	Copper (10 mg/kJ)	1.34	1.11–1.66	.004
	Vitamin B_1_ (mg/kJ)	2.15	0.21–20.27	.500
Model 2	Copper (10 mg/kJ)	1.17	1.03–1.35	.021
Model 3	Copper (10 mg/kJ)	1.25	1.08–1.50	.006

Note

Model 1: multivariate logistic regression model, adjusted for the selected nutrients. Model 2: multivariate logistic regression model, adjusted for age and body mass index. Model 3: multivariate logistic regression model, adjusted for age, body mass index, and the related background factors.

Abbreviations: CI, confidence interval; OR, odds ratio.

## 4 DISCUSSION

In this cross‐sectional study, severity of subjective forgetfulness was found to be significantly associated with a high Cu consumption in Japanese senior women. We also found differences in background characteristics such as body composition, physical and psychological symptoms, and vascular health between the two age groups, indicating that our findings could be affected by these distinctions.

The main dietary sources of Cu, an essential trace element, are seafood, nuts, and soybeans. Most (~95%) serum Cu is tightly bound to caeruloplasmin, a glycoprotein produced in the liver and related to Cu and iron metabolism, and small amounts of Cu comprise Cu bound to albumin, small peptides and amino acids, often called noncaeruloplasmin‐bound Cu or free Cu, which can reach the brain via the blood–brain barrier because of low molecular weight. Cu plays a key role in redox reactions via Cu‐dependent enzymes such as superoxide dismutase, cytochrome *c* oxidase, ceruloplasmin, dopamine beta‐hydroxylase, and peptidylglycine α‐hydroxylating monooxygenase, contributing to antioxidative defense, iron and energy metabolism, and neurotransmitter and neuropeptide synthesis. On the other hand, Cu overload leads to raised nonbound caeruloplasmin Cu, causing increased oxidative stress and cell damage (Liao et al., 2019). It is well‐known that Cu excess in Wilson’s disease and Cu deficiency in Menkes disease causes neurological disorders. Therefore, Cu levels in the brain are strictly regulated. Additionally, many studies have reported associations between Cu metabolism and neurodegenerative diseases, such as AD, Parkinson's disease, and Huntington's disease (Bagheri, Squitti, Haertlé, Siotto, & Saboury, 2018; Scheiber et al., 2014). However, there has been no consistency among studies on the relationship between Cu and these diseases, so the exact role of Cu in these diseases has not been fully elucidated.

Alzheimer’s disease is an irreversible progressive disease characterized by neurofibrillary tangles and senile plaque (β‐amyloid deposits). Significantly lowered Cu levels in the hippocampus in AD patients have been reported (Deibel, Ehmann, & Markesbery, 1996; Xu et al., 2016). A meta‐analysis found Cu depletion in several brain regions in patients with AD (Schrag, Mueller, Oyoyo, Smith, & Kirsch, 2011). In contrast, several reports have found a high accumulation of Cu in senile plaque related to amyloid formation (Lovell, Robertson, Teesdale, Campbell, & Markesbery, 1998; Miller et al., 2006). Squitti et al. (2014) showed nonbound caeruloplasmin Cu to be a predictor of the development of AD in patients with mild cognitive impairment. They also reported that serum Cu levels and nonbound ceruloplasmin Cu levels in AD patients were greater than those in a healthy control group (Squitti et al., 2018). Moreover, James et al. (2012) demonstrated that deranged Cu metabolism in AD was responsible for oxidative stress. Chen et al. (2019) also reported that Cu exposure disturbed brain function in wild‐type mice and exacerbated neurodegenerative changes in AD model mice, suggesting that Cu overload could induce mitochondrial and synaptic dysfunctions, some of the early changes seen in the course of AD (Lin et al., 2016).

Similarly, there have been several reports on the association between Cu and cognitive function. A cross‐sectional study of 188 Chinese people aged 65 years or over associated high plasma Cu levels with cognitive dysfunction (Smorgon et al., 2004). In a cohort study of 1,451 residents (629 men and 849 women) aged 60 years or over, inverse correlations between plasma Cu concentrations and long‐ and short‐time recall were observed only in women (Lam et al., 2008). Salustri et al. (2010) found that serum levels of nonbound caeruloplasmin Cu were negatively correlated with cognitive function in 64 healthy women over 50 years old. Furthermore, in a prospective study of 3,718 male and female local residents aged 65 years or over, cognitive function in those with a high intake of saturated and trans fats declined rapidly via increased Cu intake (Morris et al., 2006). An animal study also demonstrated that Cu consumption induced β‐amyloid accumulation and impairment of memory and learning in cholesterol‐fed rabbits, suggesting that Cu obstructs β‐amyloid clearance and accelerates its accumulation, caused by elevated brain cholesterol (Sparks & Schreurs, 2003). Our findings of an association between increased Cu intake and subjective forgetfulness only in senior women might indicate that changes in lipid metabolism caused by estrogen depletion modify the effects of Cu intake on cognitive function in elderly women. According to the National Health and Nutrition Survey 2017 conducted in Japan, the mean consumption of Cu gradually increased with advancing age in adult women (Ministry of Health, Labor, & Welfare, Japan, 2019). Among our study participants, the mean daily Cu intake in the senior group was significantly higher than that in the middle‐aged group, so our results might be affected by this difference.

One of the major limitations of our study was the small and narrow study population, making it difficult to generalize our findings to a wider population. Second, because of the cross‐sectional nature of our study, any causal relationship remains unclear. Moreover, we did not evaluate objective measures of cognitive function or assess the participants’ serum concentrations of Cu, nor did we investigate several influential factors on cognitive function, such as the usage of supplements and cognitive training, and on Cu metabolism including gastrointestinal disorders and renal failure. Additionally, the statistical significance of the results could have occurred by chance because of the analysis of multiple variables. Finally, the BDHQ, a method to determine the frequency of food eaten, was based on food recall and provided information only for the listed foods and beverages. Further studies should determine the exact effect of Cu intake on cognitive function.

In conclusion, in this study, high consumption of Cu was positively associated with the severity of subjective forgetfulness in Japanese senior women. Therefore, decreasing Cu intake could help ameliorate this symptom for this population.

## CONFLICT OF INTEREST

MT received an unrestricted research grant from the Kikkoman Corporation. The other authors have no conflicts of interest to disclose.

## ETHICAL REVIEW

This study was approved by the Tokyo Medical and Dental University Review Board.

## INFORMED CONSENT

Written informed consent was obtained from all study participants.

## Supporting information

Tables S1‐S4

## References

[fsn31740-bib-0001] Alzheimer’s Disease International (2015). World Alzheimer report 2015. The global impact of dementia: An analysis of prevalence, incidence, cost and trends. London, UK: Alzheimer’s Disease International Retrieved from https://www.alz.co.uk/research/worldalzheimerreport2015summary.pdf

[fsn31740-bib-0002] Bagheri, S. , Squitti, R. , Haertlé, T. , Siotto, M. , & Saboury, A. A. (2018). Role of copper in the onset of Alzheimer’s disease compared to other metals. Frontiers in Aging Neuroscience, 9, 446 10.3389/fnagi.2017.00446 29472855 PMC5810277

[fsn31740-bib-0003] Baltes, P. B. , & Lindenberger, U. (1997). Emergence of a powerful connection between sensory and cognitive functions across the adult life span: A new window to the study of cognitive aging? Psychology and Aging, 12, 12–21. 10.1037//0882-7974.12.1.12 9100264

[fsn31740-bib-0004] Betti, S. , Orsini, M. R. , Sciaky, R. , Cristini, C. , Cesa‐Bianchi, G. , & Zandonini, G. F. (2001). Attitudes towards menopause in a group of women followed in a public service for menopause counseling. Aging Clinical and Experimental Research, 13, 331–338. 10.1007/bf03353429 11695502

[fsn31740-bib-0005] Bourre, J. M. (2006a). Effects of nutrients (in food) on the structure and function of the nervous system: Update on dietary requirements for brain. Part 1: Micronutrients. The Journal of Nutrition, Health and Aging, 10, 377–385. 17066209

[fsn31740-bib-0006] Bourre, J. M. (2006b). Effects of nutrients (in food) on the structure and function of the nervous system: Update on dietary requirements for brain. Part 2: Macronutrients. The Journal of Nutrition, Health and Aging, 10, 386–399. 17066210

[fsn31740-bib-0007] Brann, D. W. , Dhandapani, K. , Wakade, C. , Mahesh, V. B. , & Khan, M. M. (2007). Neurotrophic and neuroprotective actions of estrogen: Basic mechanisms and clinical implications. Steroids, 72, 381–405. 10.1016/j.steroids.2007.02.003 17379265 PMC2048656

[fsn31740-bib-0008] Chen, C. , Jiang, X. , Li, Y. , Yu, H. , Li, S. , Zhang, Z. , … Yang, X. (2019). Low‐dose oral copper treatment changes the hippocampal phosphoproteomic profile and perturbs mitochondrial function in a mouse model of Alzheimer’s disease. Free Radical Biology and Medicine, 135, 144–156. 10.1016/j.freeradbiomed.2019.03.002 30862541

[fsn31740-bib-0009] Cole, G. M. , & Frautschy, S. A. (2010). DHA may prevent age‐related dementia. The Journal of Nutrition, 140, 869–874. 10.3945/jn.109.113910 20181786 PMC2838628

[fsn31740-bib-0010] Deibel, M. A. , Ehmann, W. D. , & Markesbery, W. R. (1996). Copper, iron, and zinc imbalances in severely degenerated brain regions in Alzheimer’s disease: Possible relation to oxidative stress. Journal of the Neurological Science, 143, 137–142. 10.1016/s0022-510x(96)00203-1 8981312

[fsn31740-bib-0011] Devi, G. , Hahn, K. , Massimi, S. , & Zhivotovskaya, E. (2005). Prevalence of memory loss complaints and other symptoms associated with the menopause transition: A community survey. Gender Medicine, 2, 255–264. 10.1016/s1550-8579(05)80055-5 16464737

[fsn31740-bib-0012] Farina, N. , Llewellyn, D. , Isaac, M. G. , & Tabet, N. (2017). Vitamin E for Alzheimer's dementia and mild cognitive impairment. Cochrane Database Systematic Review, 1, CD002854 10.1002/14651858.CD002854.pub4 PMC6464807 28128435

[fsn31740-bib-0013] Fernstrom, J. D. (1669). Can nutrient supplements modify brain function? The American Journal of Clinical Nutrition, 71, 1669S–1675S. 10.1093/ajcn/71.6.1669S 10837313

[fsn31740-bib-0014] Fraga, V. G. , Carvalho, M. D. G. , Caramelli, P. , de Sousa, L. P. , & Gomes, K. B. (2017). Resolution of inflammation, n‐3 fatty acid supplementation and Alzheimer disease: A narrative review. Journal of Neuroimmunology, 310, 111–119. 10.1016/j.jneuroim.2017.07.005 28778434

[fsn31740-bib-0015] Gholizadeh, S. , Sadatmahalleh, S. J. , & Ziaei, S. (2018). The association between estradiol levels and cognitive function in postmenopausal women. International Journal of Reproductive Biomedicine, 16, 455–458. 10.29252/ijrm.16.7.455 30234186 PMC6129375

[fsn31740-bib-0016] Gurvich, C. , Hoy, K. , Thomas, N. , & Kulkarni, J. (2018). Sex differences and the influence of sex hormones on cognition through adulthood and the aging process. Brain Sciences, 8, E163 10.3390/brainsci8090163 30154388 PMC6162653

[fsn31740-bib-0017] Hanson, B. S. , Isacsson, S. O. , Janzon, L. , & Lindell, S. E. (1989). Social network and social support influence mortality in elderly men. The prospective population study of “Men born in 1914”, Malmö, Sweden. American Journal of Epidemiology, 130, 100–111. 10.1093/oxfordjournals.aje.a115301 2787102

[fsn31740-bib-0018] Hensel, C. , Becker, M. , Düzel, S. , Demuth, I. , Norman, K. , Steinhagen‐Thiessen, E. , … Kühn, S. (2019). Influence of nutritional tyrosine on cognition and functional connectivity in healthy old humans. NeuroImage, 193, 139–145. 10.1016/j.neuroimage.2019.03.005 30853567

[fsn31740-bib-0019] Hirose, A. , Terauchi, M. , Akiyoshi, M. , Owa, Y. , Kato, K. , & Kubota, T. (2016). Subjective insomnia is associated with low sleep efficiency and fatigue in middle‐aged women. Climacteric, 19, 369–374. 10.1080/13697137.2016.1186160 27175855

[fsn31740-bib-0020] Hunter, M. , Battersby, R. , & Whitehead, M. (1986). Relationships between psychological symptoms, somatic complaints and menopausal status. Maturitas, 8, 217–228. 10.1016/0378-5122(86)90029-0 3784918

[fsn31740-bib-0021] James, S. A. , Volitakis, I. , Adlard, P. A. , Duce, J. A. , Masters, C. L. , Cherny, R. A. , & Bush, A. I. (2012). Elevated labile Cu is associated with oxidative pathology in Alzheimer disease. Free Radical Biology and Medicine, 52, 298–302. 10.1016/j.freeradbiomed.2011.10.446 22080049

[fsn31740-bib-0022] Kobayashi, S. , Honda, S. , Murakami, K. , Sasaki, S. , Okubo, H. , Hirota, N. , … Date, C. (2012). Both comprehensive and brief self‐administered diet history questionnaires satisfactorily rank nutrient intakes in Japanese adults. Journal of Epidemiology, 22, 151–159. 10.2188/jea.je20110075 22343326 PMC3798594

[fsn31740-bib-0023] Kobayashi, S. , Murakami, K. , Sasaki, S. , Okubo, H. , Hirota, N. , Notsu, A. , … Date, C. (2011). Comparison of relative validity of food group intakes estimated by comprehensive and brief‐type self‐administered diet history questionnaires against 16 d dietary records in Japanese adults. Public Health Nutrition, 14, 1200–1211. 10.1017/S1368980011000504 21477414

[fsn31740-bib-0024] Lam, P. K. , Kritz‐Silverstein, D. , Barrett‐Connor, E. , Milne, D. , Nielsen, F. , Gamst, A. , … Wingard, D. (2008). Plasma trace elements and cognitive function in older men and women: The Rancho Bernardo study. The Journal of Nutrition, Health and Aging, 12, 22–27. 10.1007/bf02982160 PMC2647138 18165841

[fsn31740-bib-0025] Liao, J. , Yang, F. , Chen, H. , Yu, W. , Han, Q. , Li, Y. , … Tang, Z. (2019). Effects of copper on oxidative stress and autophagy in hypothalamus of broilers. Ecotoxicology and Environmental Safety, 185, 109710 10.1016/j.ecoenv.2019.109710 31563750

[fsn31740-bib-0026] Lin, X. , Wei, G. , Huang, Z. , Qu, Z. , Huang, X. , Xu, H. , … Yang, X. (2016). Mitochondrial proteomic alterations caused by long‐term low‐dose copper exposure in mouse cortex. Toxicology Letters, 263, 16–25. 10.1016/j.toxlet.2016.10.009 27769873

[fsn31740-bib-0027] Lovell, M. A. , Robertson, J. D. , Teesdale, W. J. , Campbell, J. L. , & Markesbery, W. R. (1998). Copper, iron and zinc in Alzheimer’s disease senile plaques. Journal of the Neurological Sciences, 158, 47–52. 10.1016/s0022-510x(98)00092-6 9667777

[fsn31740-bib-0028] Markou, A. , Duka, T. , & Prelevic, G. M. (2005). Estrogens and brain function. Hormones, 4, 9–17. 10.14310/horm.2002.11138 16574627

[fsn31740-bib-0029] Miller, L. M. , Wang, Q. , Telivala, T. P. , Smith, R. J. , Lanzirotti, A. , & Miklossy, J. (2006). Synchrotron‐based infrared and X‐ray imaging shows focalized accumulation of Cu and Zn co‐localized with beta‐amyloid deposits in Alzheimer’s disease. Journal of Structural Biology, 155, 30–37. 10.1016/j.jsb.2005.09.004 16325427

[fsn31740-bib-0030] Ministry of Health, Labor and Welfare, Japan (2019). National Health and Nutrition Survey 2017. Retrieved from https://www.mhlw.go.jp/content/10904750/000351576.pdf

[fsn31740-bib-0031] Morris, M. C. , Evans, D. A. , Tangney, C. C. , Bienias, J. L. , Schneider, J. A. , Wilson, R. S. , & Scherr, P. A. (2006). Dietary copper and high saturated and trans fat intakes associated with cognitive decline. Archives of Neurology, 63, 1085–1088. 10.1001/archneur.63.8.1085 16908733

[fsn31740-bib-0032] Park, D. C. , Smith, A. D. , Lautenschlager, G. , Earles, J. L. , Frieske, D. , Zwahr, M. , & Gaines, C. L. (1996). Mediators of long‐term memory performance across the life span. Psychology and Aging, 11, 621–637. 10.1037//0882-7974.11.4.621 9000294

[fsn31740-bib-0033] Previc, F. H. (1999). Dopamine and the origins of human intelligence. Brain and Cognition, 41, 299–350. 10.1006/brcg.1999.1129 10585240

[fsn31740-bib-0034] Rosenberg, L. , & Park, S. (2002). Verbal and spatial functions across the menstrual cycle in healthy young women. Psychoneuroendocrinology, 27, 835–841. 10.1016/s0306-4530(01)00083-x 12183218

[fsn31740-bib-0035] Salustri, C. , Barbati, G. , Ghidoni, R. , Quintiliani, L. , Ciappina, S. , Binetti, G. , & Squitti, R. (2010). Is cognitive function linked to serum free copper levels? A cohort study in a normal population. Clinical Neurophysiology, 121, 502–507. 10.1016/j.clinph.2009.11.090 20097602

[fsn31740-bib-0036] Scheiber, I. F. , Mercer, J. F. , & Dringen, R. (2014). Metabolism and functions of copper in brain. Progress in Neurobiology, 116, 33–57. 10.1016/j.pneurobio.2014.01.002 24440710

[fsn31740-bib-0037] Schrag, M. , Mueller, C. , Oyoyo, U. , Smith, M. A. , & Kirsch, W. M. (2011). Iron, zinc and copper in the Alzheimer’s disease brain: A quantitative meta‐analysis. Some insight on the influence of citation bias on scientific opinion. Progress in Neurobiology, 94, 296–306. 10.1016/j.pneurobio.2011.05.001 21600264 PMC3134620

[fsn31740-bib-0038] Smorgon, C. , Mari, E. , Atti, A. R. , Dalla Nora, E. , Zamboni, P. F. , Calzoni, F. , … Fellin, R. (2004). Trace elements and cognitive impairment: An elderly cohort study. Archives of Gerontology and Geriatrics. Suppl, 9, 393–402. 10.1016/j.archger.2004.04.050 15207438

[fsn31740-bib-0039] Sparks, D. L. , & Schreurs, B. G. (2003). Trace amounts of copper in water induce beta‐amyloid plaques and learning deficits in a rabbit model of Alzheimer’s disease. Proceedings of the National Academy of Sciences of the United States of America, 100, 11065–11069. 10.1073/pnas.1832769100 12920183 PMC196927

[fsn31740-bib-0040] Squitti, R. , Ghidoni, R. , Simonelli, I. , Ivanova, I. D. , Colabufo, N. A. , Zuin, M. , … Siotto, M. (2018). Copper dyshomeostasis in Wilson disease and Alzheimer's disease as shown by serum and urine copper indicators. Journal of Trace Elements in Medicine and Biology, 45, 181–188. 10.1016/j.jtemb.2017.11.005 29173477

[fsn31740-bib-0041] Squitti, R. , Ghidoni, R. , Siotto, M. , Ventriglia, M. , Benussi, L. , Paterlini, A. , … Pasqualetti, P. (2014). Value of serum nonceruloplasmin copper for prediction of mild cognitive impairment conversion to Alzheimer disease. Annals of Neurology, 75, 574–580. 10.1002/ana.24136 24623259

[fsn31740-bib-0042] Sullivan Mitchell, E. , & Fugate Woods, N. (2001). Midlife women’s attributions about perceived memory changes: Observations from the Seattle Midlife Women’s Health Study. Journal of Women’s Health & Gender‐Based Medicine, 10, 351–362. 10.1089/152460901750269670 11445026

[fsn31740-bib-0043] Terauchi, M. , Hiramitsu, S. , Akiyoshi, M. , Owa, Y. , Kato, K. , Obayashi, S. , … Kubota, T. (2012). Associations between anxiety, depression and insomnia in peri‐ and post‐menopausal women. Maturitas, 72, 61–65. 10.1016/j.maturitas.2012.01.014 22326659

[fsn31740-bib-0044] Terauchi, M. , Hirose, A. , Akiyoshi, M. , Kato, K. , & Miyasaka, N. (2017). Feelings of unattractiveness in peri‐ and postmenopausal women are associated with depressed mood, poor memory and unsatisfactory sexual relationships. Climacteric, 20, 228–232. 10.1080/13697137.2017.1293647 28285543

[fsn31740-bib-0045] Terauchi, M. , Hirose, A. , Akiyoshi, M. , Owa, Y. , Kato, K. , & Kubota, T. (2015). Prevalence and predictors of storage lower urinary tract symptoms in perimenopausal and postmenopausal women attending a menopause clinic. Menopause, 22, 1084–1090. 10.1097/GME.0000000000000432 25756698

[fsn31740-bib-0046] Terauchi, M. , Obayashi, S. , Akiyoshi, M. , Kato, K. , Matsushima, E. , & Kubota, T. (2010). Insomnia in Japanese peri‐ and postmenopausal women. Climacteric, 13, 479–486. 10.3109/13697130903353478 19886814

[fsn31740-bib-0047] Terauchi, M. , Obayashi, S. , Akiyoshi, M. , Kato, K. , Matsushima, E. , & Kubota, T. (2011). Effects of oral estrogen and hypnotics on Japanese peri‐ and postmenopausal women with sleep disturbance. Journal of Obstetrics and Gynaecology Research, 37, 741–749. 10.1111/j.1447-0756.2010.01424.x 21395896

[fsn31740-bib-0048] van Keep, P. A. , & Kellerhals, J. M. (1974). The impact of socio‐cultural factors on symptom formation. Some results of a study on ageing women in Switzerland. Psychotherapy and Psychosomatics, 23, 251–263. 10.1159/000286648 4416302

[fsn31740-bib-0049] Viña, J. , & Lloret, A. (2010). Why women have more Alzheimer’s disease than men: Gender and mitochondrial toxicity of amyloid‐beta peptide. Journal of Alzheimer’s Disease, 20(Suppl 2), S527–S533. 10.3233/JAD-2010-100501 20442496

[fsn31740-bib-0050] Xu, J. , Begley, P. , Church, S. J. , Patassini, S. , McHarg, S. , Kureishy, N. , … Cooper, G. J. S. (2016). Elevation of brain glucose and polyol‐pathway intermediates with accompanying brain‐copper deficiency in patients with Alzheimer’s disease: Metabolic basis for dementia. Scientific Reports, 6, 7524 10.1038/srep27524 PMC4899713 27276998

[fsn31740-bib-0051] Zigmond, A. S. , & Snaith, R. P. (1983). The hospital anxiety and depression scale. Acta Psychiatrica Scandinavica, 67, 361–370. 10.1111/j.1600-0447.1983.tb09716.x 6880820

