# Supplementary material for: Severity of subjective forgetfulness is associated with high dietary intake of copper in Japanese senior women: A cross‐sectional study
Source: Food Sci Nutr. 2020 Jul 1;8(8):4422–31. doi: 10.1002/fsn3.1740 (PMC7455963; doi:10.1002/fsn3.1740)
Supplement: Supplementary file 1 — Tables S1‐S4 [file FSN3-8-4422-s001.docx]

Table S1. Menopausal Health-Related Quality of Life Questionnaire.

Table S2. The 43 major nutrients assessed with the BDHQ.

BDHQ, brief-type self-administered diet history questionnaire

Table S3. Comparison of the characteristics of the middle-aged and senior female participants between the severity categories of subjective forgetfulness.

Values are mean (standard deviation) or percentage. NA, not available, ^†^Mann–Whitney test, ^‡^chi-squared test.
